# Editors’ Book Table

**Published:** 1872-01

**Authors:** 


### Editors' Book Table.

[NOTE. — All works reviewed in the columns of the CHICAGO MEDICAL JOURNAL may be found in the extensive stock of W. B. KEEN, COOKE & CO. whose catalogue of Medical Books will be sent to any address upon request.]

*Rindfleisch's Text-Book of Pathological Histology. An Introduction to the Study of Pathological Anatomy.* By DR. EDWARD RINDFLEISCH, O. O. Professor of Pathological Anatomy in Bonn. Translated from the Second German Edition, by WM. C. KLOMAN, M.D., assisted by F. T. MILES, M.D., Prof. of Anatomy, University of Maryland, etc., etc. Containing 208 Elaborately Executed Microscopical Illustrations. By S. C. Griggs & Co. One volume, octavo. Price, cloth, $6.00; sheep, $7.00.

CONTENTS.

Introduction, Author's and Editor's Prefaces.

*General Part.*—1. Decomposition and Degeneration of Tissues; 2. Pathological New Formations.

*Special Part.*—1. Anomalies of the Blood, and the Places of its Formation, especially of the Spleen and Lymphatic Glands; 2. Anomalies of the Circulatory Apparatus; 3. Anomalies of Serous Membranes; 4. Anomalies of the Skin; 5. Anomalies of Mucous Membranes; 6. Anomalies of the Lung; 7. Anomalies of the Liver; 8. Anomalies of the Kidney; 9. Anomalies of the Ovaries; 10. Anomalies of the Testicles; 11. Anomalies of the Mammæ; 12. Anomalies of the Prostate Gland; 13. Anomalies of the Salivary Glands; 14. Anomalies of the Thyroid Gland; 15. Anomalies of the Suprarenal Capsules; 16. Anomalies of the Osseous System ; 17 ; Anomalies of the Nervous System ; 18. Anomalies of the Muscular System.

Index and Bibliography.

Prof. Rindfleisch's Text Book of Pathological Histology, so justly celebrated in Germany, where it is considered the most complete and thorough work of its kind, having passed rapidly to a second edition, is also very highly valued and commended by German medical scholars in this country, many of whom are not only familiar with the book, but with the author's great reputation as a teacher and professor of this branch of medical study.

The translators are both gentlemen, who, by their past education, have been peculiarly fitted for the task of translating the work. Dr. Kloman from early life has been familiar with the German language, while Prof. Miles has made the subject one of special study, both gentlemen being also practical microscopists. The publishers, therefore, offer a translation of this truly valuable work to the Medical Profession in the United States, feeling the utmost confidence that in both manner and style it will prove acceptable to them. In their preface, the translators say : " In presenting the English reading portion of the Medical Profession with a translation of the valuable work of Professor Rindfleisch, the translators scarcely deem an apology necessary. The merits of the book itself, and the fact that it fills an unoccupied gap in our most recent literature upon the subject of Pathological Histology, was judged to be an ample incentive for undertaking the labor of the translation. The work of VIRCHOW translated by CHANCE, is, in many points, antiquated ; and the more recent work of BILLROTH, translated by HACKLEY, occupies the ground but partially, and is professedly a work of Surgical Pathology.

The work is translated and published in this country by special arrangement with the author.

This book is indispensable to the reading physician.

*Electricity in its Relations to Practical Medicine.* By DR. MORITZ MEYER, Royal Counselor of Health, etc. Second revised and corrected American Edition, translated from Third German Edition, with notes and additions, by WM. A. HAMMOND, M.D., Professor of Diseases of the Mind and Nervous System and of Clinical Medicine, in the Bellevue Hospital Medical College, etc. New York : D. Appleton & Co. 1872.

*Anæsthesia, Hospitalism, Hermaphroditism, and a Proposal to Stamp out Small Pox and other Cutaneous Diseases.* By SIR JAMES Y. SIMPSON, Bart., M.D., D. C. L., late Professor of Midwifery in the University of Edinburgh. Edited by SIR W. G. SIMPSON, Bart., B. A., Scholar of Gonville and Caius College, Cambridge. New York : D. Appleton & Co. 1872.

*A Handbook of Therapeutics.* By Sydney Ringer, M.D., Prof. of Therapeutics in University College, etc. New York: William Wood & Co. 1871.

*Putnam's Handybook Series. Eating and Drinking;* A Popular Manual of Food and Diet in Health and Disease. By Geo. M. Beard, M.D. New York: G. P. Putnam & Sons. 1871.

*Putnam's Handybook Series. Stimulants and Narcotics;* Medically, Philosophically and Morally considered. By Geo. M. Beard, M.D. New York: G. P. Putnam & Sons. 1871.

*Neuralgia, and the Diseases that Resemble it.* By Francis E. Anstie, M.D. New York: D. Appleton & Co. 1872.

Dr. Anstie is too well known a writer to need introduction to our readers. The first part of his book considers pure neuralgia, its nature, complications and treatment. The latter part investigates what he considers as *simulating* affections, such as the pains of locomotor ataxy, alcoholism, syphilis, "spinal irritation," etc.

PAMPHLETS, ETC., RECEIVED.

*The Half-Yearly Abstract of the Medical Sciences.* Being a digest of British and Continental Medicine, and of the progress of Medicine and the collateral Sciences. Edited by William Domett Stone, M.D., F. R. C. S. (Exam.) Volume LIV. January, 1872. Philadelphia: Henry C. Lea.

*Report of the Chicago Relief and Aid Society to the Common Council of the City of Chicago.*

*American Association for the Cure of Inebriates. Proceedings of the Second Meeting, held in New York, November 14th and 15th,* 1871. Published by order of the Association.

*Annual Report of the Superintendent and Physician* of the New York State Inebriate Asylum, Binghampton, N. Y., for the year 1871.

*Transactions of the Iowa State Medical Society.* Compiled and Arranged by A. G. Field, M.D., S. B. Thrall, M.D., and J. Williamson, M.D., Committee on Publication.

*The Publishers' and Stationers' Weekly Trade Circular.* F. Leypoldt, Editor and Publisher, 712 Broadway, New York.

